# Long noncoding RNA CASC7 inhibits the proliferation and migration of papillary thyroid cancer cells by inhibiting miR-34a-5p

**DOI:** 10.1186/s12576-021-00793-2

**Published:** 2021-03-11

**Authors:** Wencong Sun, Detao Yin

**Affiliations:** 1 Department of Thyroid Surgery, The First Affiliated Hospital of Zhengzhou University, No.1 Jianshe Road, Zhengzhou, 450052 Henan People’s Republic of China; 2 Department of Thyroid Surgery, Henan Provincial People’s Hospital, People’s Hospital of Zhengzhou University, No.7 Weiwu Road, Zhengzhou, 450003 Henan People’s Republic of China; 3 Key Discipline Laboratory of Clinical Medicine of Henan, Daxue Road, Zhengzhou, 450050 Henan People’s Republic of China

**Keywords:** CASC7, miR-34a-5p, TP73, Thyroid cancer

## Abstract

Long noncoding RNAs (lncRNAs) play an essential role in the progression of papillary thyroid cancer (PTC). However, the expression and function of lncRNA cancer susceptibility candidate 7 (CASC7) in PTC remain unknown. The purpose of this study was to investigate the role and molecular mechanism of CASC7 in regulating PTC cell behavior. The expression of CASC7, miR-34a-5p, and tumor protein P73 (TP73) was determined by qRT-PCR and western blot. Cell proliferation was examined by MTT assay. Cell apoptosis was assessed by flow cytometry following Annexin V and PI staining. Cell migration was determined by Transwell migration assay. The interaction between miR-34a-5p and CASC7 or TP73 was examined by luciferase reporter assay. CASC7 and TP73 expression were significantly lower, whereas miR-34a-5p expression was higher in PTC tissues than the adjacent normal tissues. Furthermore, CASC7 overexpression inhibited cell proliferation and migration, whereas facilitated cell apoptosis in human PTC cell lines (K1 and TPC-1). Mechanistically, CASC7 acted as a sponge of miR-34a-5p to upregulate TP73 expression. Moreover, miR-34a-5p mimic transfection could abate the CASC7-regulated PTC cell proliferation, migration, and apoptosis. Collectively, CASC7 inhibited the proliferation and migration of PTC cells by sponging miR-34a-5p to upregulate TP73 expression.

## Background

Thyroid cancer (TC) is the most common endocrine malignancy, accounting for about 1% of all human malignant neoplasms [1, 2]. TC can be classified into papillary thyroid cancer (PTC), follicular carcinoma, undifferentiated carcinoma, and medullary carcinoma according to the specific histological features [3]. PTC is the most prevalent form of thyroid malignancy, accounting for more than 80% of TC [4]. PTC has a relatively good prognosis, but there are still cases (< 10%) that present with tumors exhibiting aggressive characteristics [5]. Therefore, in-depth exploration of the mechanism of PTC occurrence and development has significant clinical implications for the diagnosis and treatment of PTC.

Long noncoding RNAs (lncRNAs) are a class of RNA molecules with a length of more than 200 nucleotides that participate in various physiological or pathological processes [6]. LncRNAs have been a hotspot in cancer research. Considerable studies have confirmed that the abnormal expression of lncRNAs is closely related to the initiation and development of various cancers, including PTC [7, 8]. Certain lncRNAs are lowly expressed in PTC tissues and have tumor-suppressive function in PTC, for example, H19 [9], maternally expressed 3 [10], and BRAF-activated non-protein coding RNA [11]. In contrast, certain lncRNAs that are highly expressed in PTC tissues to exert oncogenic function in PTC, for example, copy number amplified long noncoding RNA in papillary thyroid cancer 1 [12], lncRNA-activated by TGF-beta [13], and nuclear paraspeckle assembly transcript 1 [14]. The above-mentioned findings indicate that lncRNAs are important for understanding the molecular biology of PTC progression.

LncRNA CASC7 (cancer susceptibility candidate 7) is a ∼9.3 kb lncRNA with tumor-suppressive roles in some cancers. For instance, Zhang et al*.* demonstrated that CASC7 is lowly expressed in colorectal cancer tissues and suppresses colorectal cancer cell proliferation and migration via inhibiting microRNA (miR)-21 [15]. More recently, Gong et al*.* reported that CASC7 is downregulated in glioma tissues and inhibits the progression of glioma via regulating Wnt/β-catenin signaling pathway [16]. However, the expression and function of CASC7 in other cancers remain largely undefined, and the present study was designed to explore its role and molecular mechanism in PTC.

## Materials and methods

### Human sample collection

Thirty PTC tissues and adjacent normal tissues were obtained from patients who underwent surgical treatment for PTC at our hospital. Samples were stored at − 80 °C until use. The diagnosis of PTC was confirmed histopathologically by an experienced pathologist. The study was approved by the Ethics committee of First Affiliated Hospital of Zhengzhou University, and all participants have signed a written informed consent for using the specimens.

### Cell culture

Human PTC cell lines (K1 and TPC-1) were purchased from BeNa Culture Collection (Beijing, China). Cells were maintained in high-glucose Dulbecco’s modified Eagle’s medium (DMEM; Gibco, Thermo Fisher Scientific, Inc., Waltham, MA, USA) supplemented with 10% fetal bovine serum (FBS; Gibco), glutamine, and sodium pyruvate in an incubator at 37 °C with 5% CO_2_.

### Cell transfection

To overexpress CASC7, the full-length gene sequence of CASC7 was synthesized and subcloned into a pcDNA3.1 vector (Invitrogen, Thermo Fisher Scientific, Inc.), generating pcDNA3.1-CASC7. The empty pcDNA3.1 vector was used as the control. The short hairpin RNAs (shRNAs) against CASC7 (sh-CASC7), scrambled oligonucleotides (sh-NC), miR-34a-5p mimic, mimic negative control (NC), miR-34a-5p inhibitor, and inhibitor NC were purchased from GenePharma (Shanghai, China). For transfection, K1 and TPC-1 cells in the logarithmic growth phase were seeded in 6-well cell culture plates at a density of 5 × 10^3^/mL and incubated overnight at 37 °C. When the cells reached a confluency of 70 ~ 80%, cells were transfected with these vectors and/or miRNA mimic/inhibitor using Lipofectamine 2000 (Invitrogen) according to the manufacturer’s instructions. Following 48 h of transfection, cells were harvested and processed for further analysis.

### Cell viability and apoptosis assay

For cell viability assay, cells were plated in 96-well cell culture plates at a density of 3 × 10^5^ cells/mL with 100 µL culture medium. After incubation for 24 h, 20 μL of MTT (5 mg/mL) was added to each well and the cells were incubated for an additional 4 h. Subsequently, the culture medium was removed and 150 µL of DMSO was added to each well. The absorbance in each well was measured at 490 nm using a microplate reader.

Cell apoptosis was measured by flow cytometry using the Annexin V-FITC/PI Apoptosis Kit (KeyGENE Biotechnology, Nanjing, China) according to the manufacturer’s instructions. The resulting fluorescence was measured by flow cytometry using the Becton Dickinson FACSCalibur (Becton Dickinson, Franklin Lakes, NJ, USA).

### Transwell migration assay

After different transfection treatments, K1 and TPC-1 cells were resuspended in 100 μL serum-free medium (5 × 10^4^ cells) was added into the upper chambers of Transwell inserts (8.0-μm pore size) for migration (without Matrigel) assays. The lower chambers were filled with 500 μL DMEM medium containing 10% FBS. After 24 h, the bottom surface of the membrane was fixed in 10% formaldehyde solution, followed by 1% crystal violet staining for 2 h. The number of migrated cells was calculated and cells were imaged under a microscope from five random fields.

### Quantitative real-time PCR (qRT-PCR)

Total RNA was extracted from tissues or cell lines using RNAisoPlus Kit (Takara, Dalian, China). cDNA synthesis was performed with 1 ~ 2 µg of RNA using iScript First Strand cDNA synthesis kit (Bio-Rad, Hercules, CA, USA). After reverse transcription, the expression of CASC7 and tumor protein P73 (TP73) were detected using the SYBR premix (Takara) whereas the expression of miR-34a-5p was using the miRNA qRT-PCR kit (GeneCopoeia, Rockville, MD, USA) in Applied Biosystems 7500 PCR system (Applied Biosystems, Foster, CA, USA). U6 was employed as the endogenous control for miR-34a-5p and GAPDH for CASC7 and TP73. The primers were synthesized by Shanghai Bioengineering Co. (Shanghai, China).

### Western blot

Total protein was extracted from the cells in RIPA lysis buffer (Beyotime, Shanghai, China). Then equal protein (20 μg) from cell lysates was separated by 10% SDS-PAGE gels and electro-transferred onto PVDF membranes (Millipore, Billerica, MA, USA). The membranes were then incubated with the primary antibodies against TP73 (1:1000; Abcam, Cambridge, MA, USA) and GAPDH (1:2500; Abcam) overnight at 4 °C, followed by the horseradish peroxidase (HRP)-conjugated secondary antibodies (1:2000; Santa Cruz Biotechnology, Dallas, TX, USA). Detection was performed using an enhanced chemiluminescence (ECL) detection kit (Millipore).

### Luciferase reporter assay

The recombination luciferase plasmids containing the wild-type (WT) or mutant (Mut) gene sequences of CASC7 or TP73 3′-UTR region with miR-34a-5p binding site were constructed from Genecopoeia company (Rockville, Md, USA). K1 cells were cotransfected with the luciferase construct along with miR-34a-5p mimic and mimic NC. After 48 h of transfection, the luciferase activity was examined using the luciferase assay kit (Promega, Madison, WI, USA) according to the manufacturer’s instructions.

### Statistical analysis

Statistical analysis was performed using SPSS version 20.0 (IBM, Chicago, IL, USA). The unpaired Student’s *t* test and one-way analysis of variance were used to analyze differences between two or more groups, respectively. *P* < 0.05 was considered to have a statistically significant difference.

## Results

### CASC7 overexpression inhibited PTC cell proliferation and migration, and induced PTC cell apoptosis

**Fig. 1:**
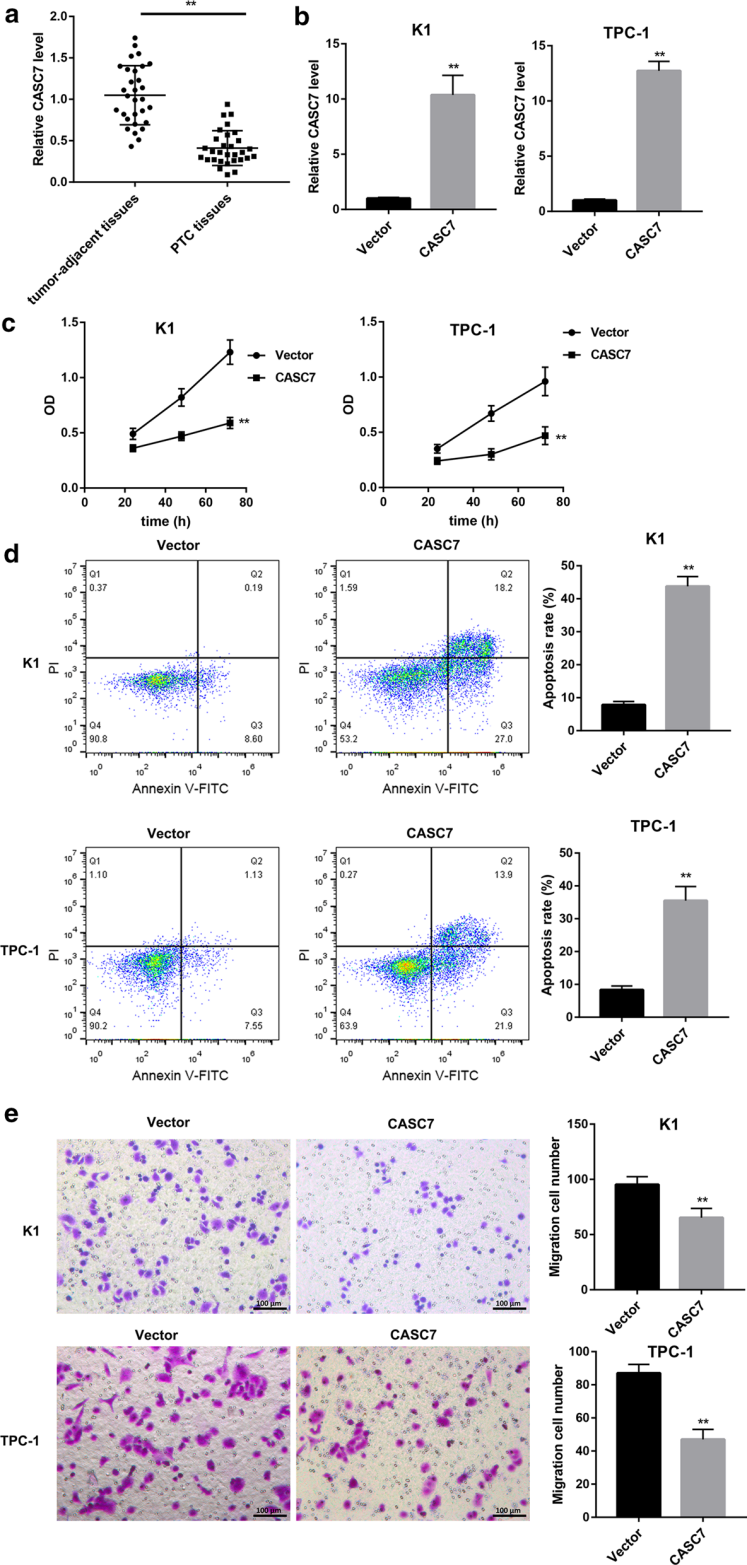
CASC7 overexpression inhibited PTC cell proliferation and migration, and induced cell apoptosis. **a** qRT-PCR analysis of CASC7 expression in PTC tissues (*n* = 30) and adjacent normal tissues (*n* = 30). **b** CASC7 expression determined by qRT-PCR analysis, **c** cell viability assessed by MTT assay, **d** cell apoptosis determined by flow cytometry following Annexin V and PI staining, and **e** cell migration assessed by Transwell migration assay in human PTC cell lines (K1 and TPC-1) transfected with the CASC7 overexpression vector and empty vector. **P < 0.01 vs. Tumor-adjacent tissues or Vector. Data are expressed as the mean ± standard deviation (*n* = 3).

qRT-PCR analysis showed that CASC7 expression was significantly lower in most PTC tissues than that in adjacent normal tissues (Fig. 1a). Furthermore, CASC7 expression showed a downward trend with the PTC stage progression (Additional file 1: Figure S1). To examine the functional role of CASC7 in regulating PTC cell behavior, we overexpressed CASC7 in two human PTC cell lines (K1 and TPC-1). The overexpression efficiency of CASC7 was confirmed by qRT-PCR analysis in both cells (Fig. 1b). CASC7 overexpression led to impaired cell growth (Fig. 1c) and enhanced cell apoptosis (Fig. 1d). Furthermore, Transwell migration assay demonstrated that the number of migrated cells in PTC cells following CASC7 overexpression was notably less than that in those transfected with empty vector (Fig. 1e).

### CASC7 negatively regulated miR-34a-5p expression, whereas positively regulated TP73 expression

**Fig. 2:**
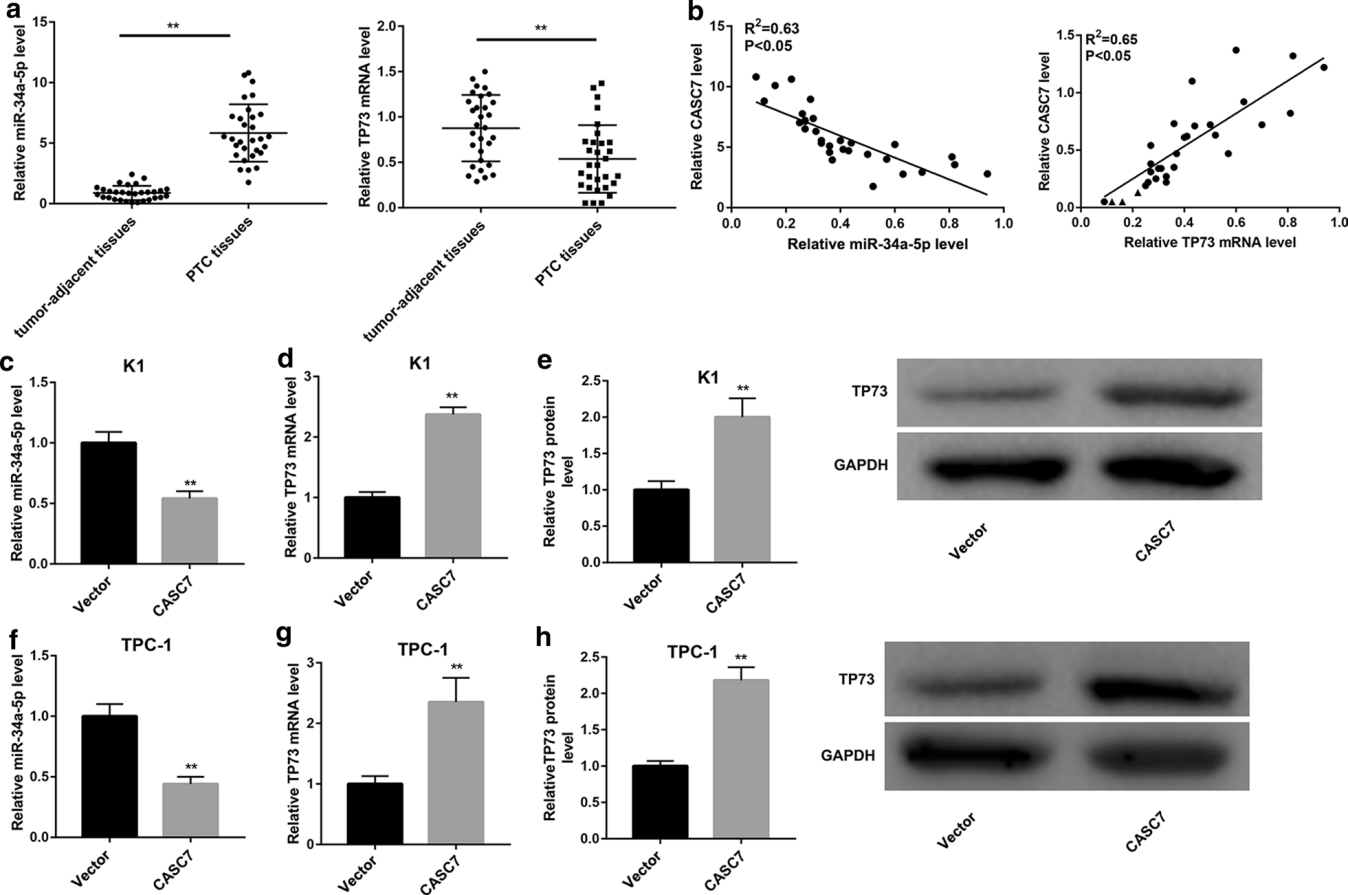
CASC7 negatively regulated miR-34a-5p expression, whereas positively regulated TP73 expression. **a** qRT-PCR analysis of miR-34a-5p and TP73 expression in PTC tissues (*n* = 30) and adjacent normal tissues (*n* = 30). **b** Negative correlation between CASC7 expression and miR-34a-5p expression, and positive correlation between CASC7 expression and TP73 mRNA expression in PTC tissues (*n* = 30). **c**, **f** miR-34a-5p expression determined by qRT-PCR analysis, **d**, **g** TP73 mRNA expression determined by qRT-PCR analysis, and **e**, **h** TP73 protein level determined by western blot in human PTC cell lines (K1 and TPC-1) transfected with the CASC7 overexpression vector and empty vector. **P < 0.01 vs. Tumor-adjacent tissues or Vector. Data are expressed as the mean ± standard deviation (*n* = 3).

miR-34a-5p expression was notably higher, whereas TP73 mRNA expression was lower in most PTC tissues when compared with the adjacent normal tissues (Fig. 2a). Furthermore, CASC7 expression was inversely correlated with miR-34a-5p expression, whereas positively correlated with TP73 mRNA expression in PTC tissues (Fig. 2b). Then, we evaluated the effect of CASC7 overexpression on expression of miR-34a-5p and TP73. qRT-PCR and western blot analyses showed that CASC7 overexpression noticeably downregulated miR-34a-5p expression in both K1 and TPC-1 cells (Fig. 2c, f). Conversely, CASC7 overexpression resulted in a significant upregulation of TP73 expression, both at mRNA (Fig. 2d, g) and protein (Fig. 2e, h) levels. On the contrary, CASC7 silencing notably decreased mRNA and protein levels of TP73 in both cells (Additional file 2: Figure S2A–D).

### CASC7 acted as a sponge of miR-34a-5p to upregulate TP73 expression

**Fig. 3:**
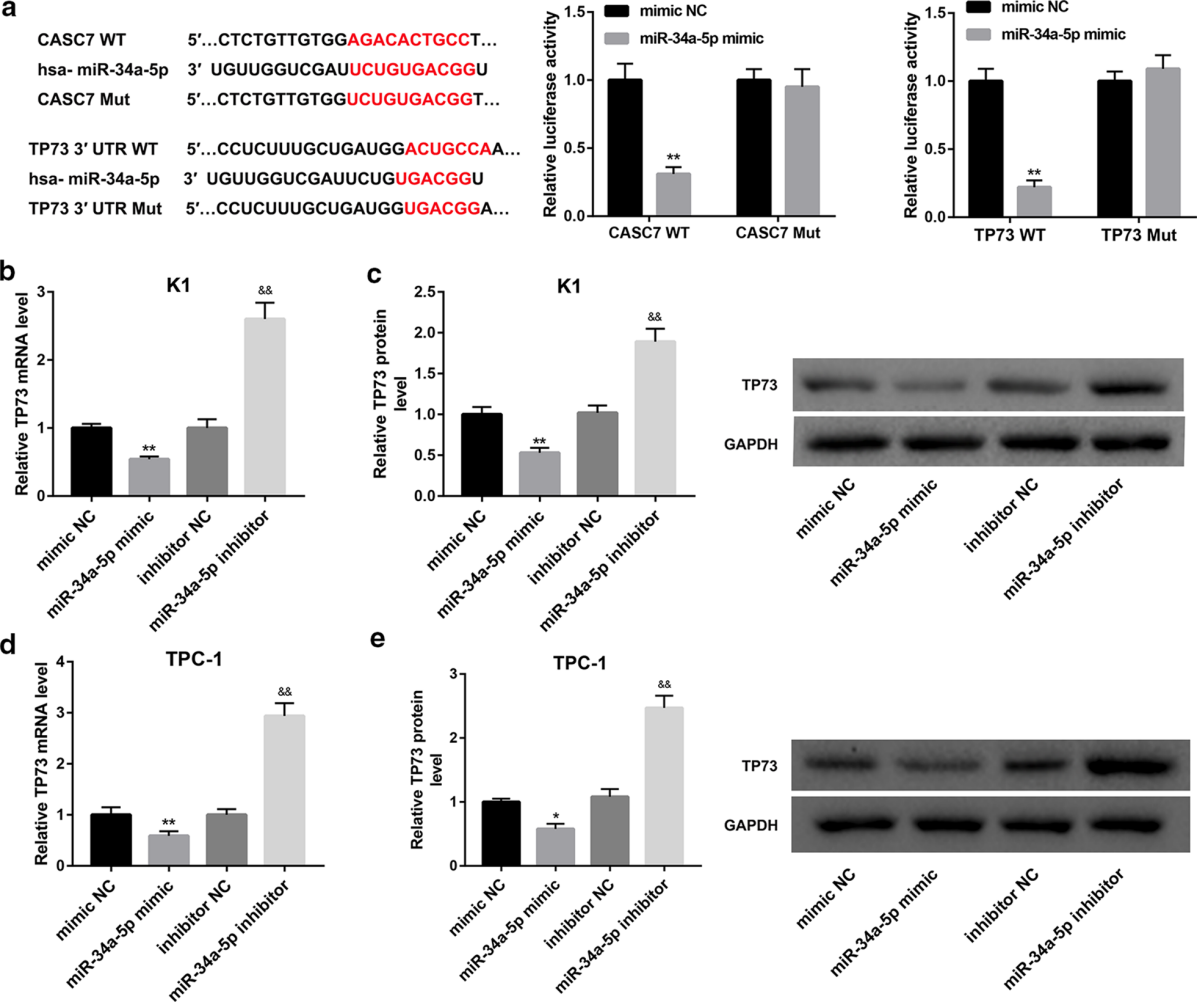
TP73 was a target of miR-34a-5p. **a** Prediction of target relationship between miR-34a-5p and CASC7 or TP73. Luciferase reporter assay was performed to evaluate the interaction ability between miR-34a-5p and CASC7 or TP73 3′-UTR. **b**, **d** TP73 mRNA expression determined by qRT-PCR analysis, and **c**, **e** TP73 protein level determined by western blot in human PTC cell lines (K1 and TPC-1) co-transfected with miR-34a-5p mimic/mimic NC and miR-34a-5p inhibitor/inhibitor NC. *P < 0.05, **P < 0.01 vs. mimic NC; ^&&^*P* < 0.01 vs. inhibitor NC. Data are expressed as the mean ± standard deviation (*n* = 3).

The results of bioinformatics analysis using the prediction tools miRanda (http://www.microrna.org/) showed that CASC7 harbors putative binding sites of miR-34a-5p, which was predicted to target the 3′-UTR of TP73 using TargetScan (http://www.targetscan.org/vert_72/) (Fig. 3a). Accordingly, we investigated the interaction between miR-34a-5p and CASC7 as well as miR-34a-5p and TP73. Luciferase reporter assay showed that miR-34a-5p mimic transfection significantly inhibited luciferase activity in the CASC7 WT but not CASC7 Mut group, indicating the direct interaction between CASC7 and miR-34a-5p. Furthermore, the miR-34a-5p mimic notably repressed luciferase activity in cells co-transfected with TP73-WT reporter, whereas had no obvious effect on the luciferase activity in TP73-Mut group, verifying TP73 was a direct target of miR-34a-5p (Fig. 3a).

Moreover, TP73 mRNA and protein levels were significantly downregulated in both K1 and TPC-1 cells following introduction of miR-34a-5p mimic. In contrast, inhibition of miR-34a-5p by miR-34a-5p inhibitor transfection markedly upregulated TP73 mRNA and protein levels in both cells (Fig. 3b–e).

**Fig. 4:**
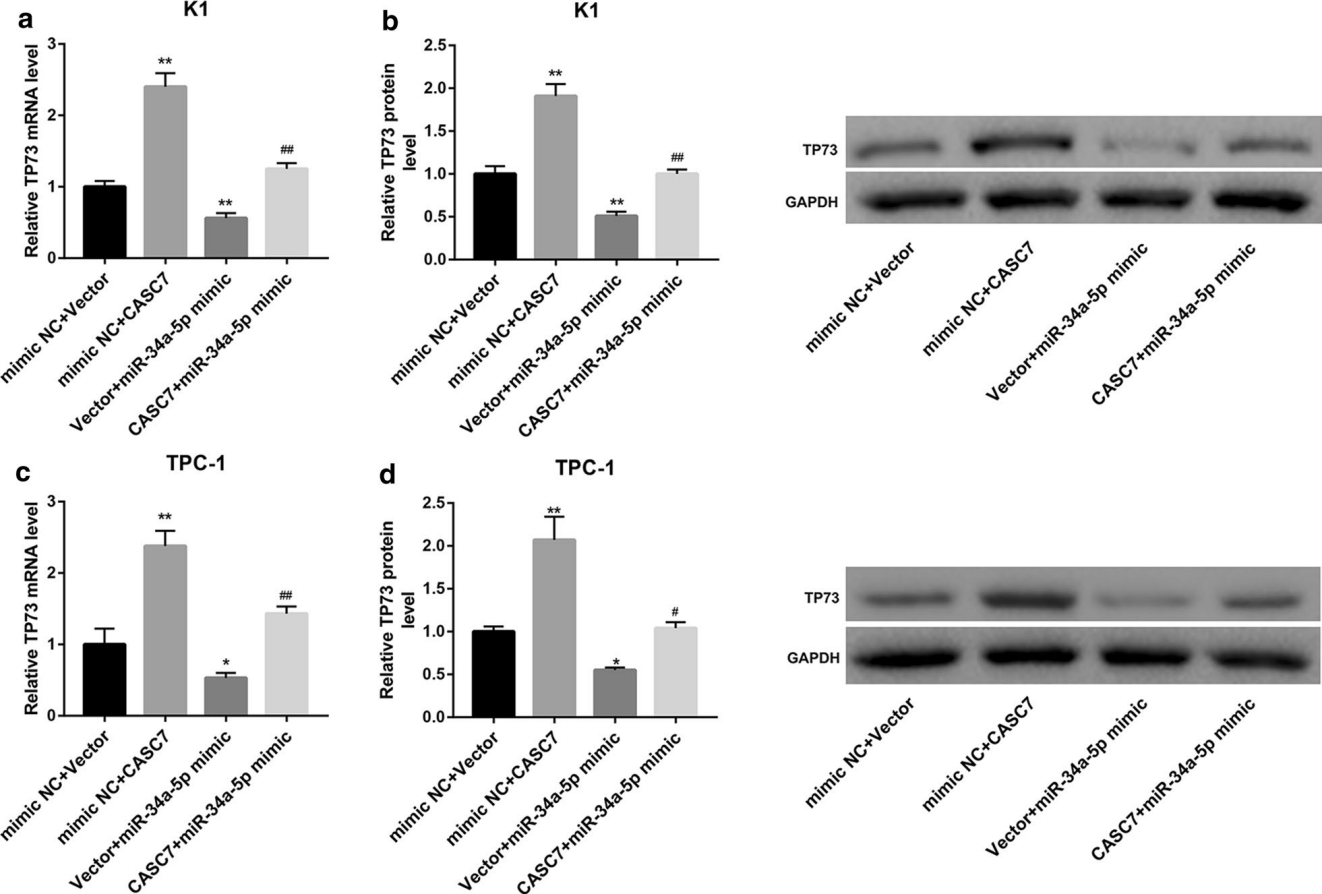
CASC7 overexpression attenuated the miR-34a-5p mimic-mediated inhibition of TP73 expression. **a**, **c** TP73 mRNA expression determined by qRT-PCR analysis, and **b**, **d** TP73 protein level determined by western blot in human PTC cell lines (K1 and TPC-1) co-transfected with miR-34a-5p mimic/mimic NC and CASC7 overexpression vector/empty vector. *P < 0.05, **P < 0.01 vs. mimic NC + Vector; ^#^*P* < 0.05, ^##^*P* < 0.01 vs. mimic NC + CASC7 or Vector + miR-34a-5p mimic. Data are expressed as the mean ± standard deviation (*n* = 3)

Importantly, CASC7 overexpression led to a notable upregulation of TP73 mRNA and protein levels in both cells. We also found that the miR-34a-5p mimic-mediated inhibition of TP73 expression could be abrogated by CASC7 overexpression (Fig. 4a–d). Taken together, these data indicated that CASC7 could function as a sponge of miR-34a-5p to increase TP73 expression.

### CASC7-regulated PTC cell behavior through sponging miR-34a-5p

**Fig. 5:**
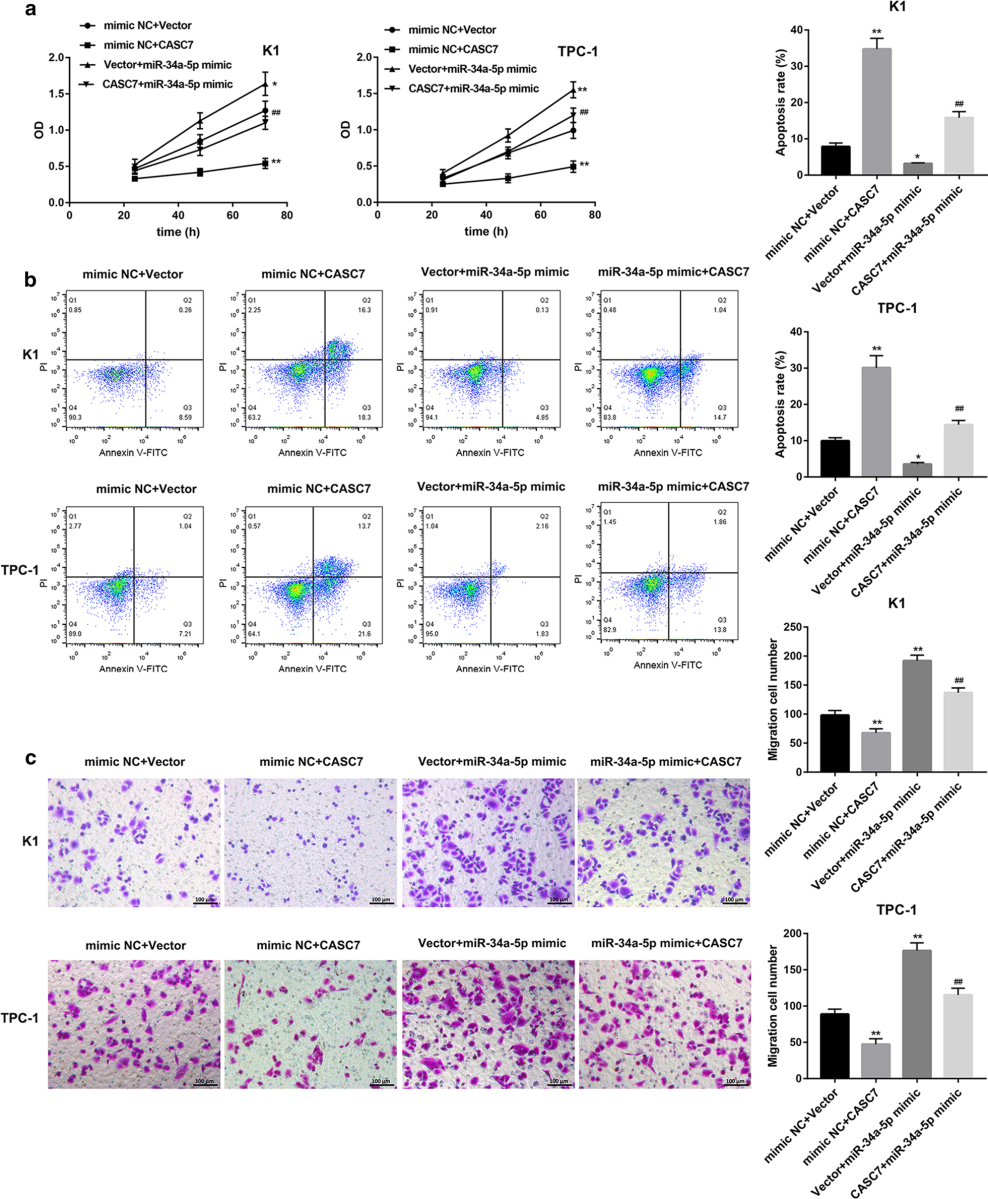
CASC7-regulated PTC cell behavior through sponging miR-34a-5p. **a** Cell viability assessed by MTT assay, **b** cell apoptosis determined by flow cytometry following Annexin V and PI staining, and **c** cell migration assessed by Transwell migration assay in human PTC cell lines (K1 and TPC-1) co-transfected with miR-34a-5p mimic/mimic NC and CASC7 overexpression vector/empty vector. *P < 0.05, **P < 0.01 vs. mimic NC + Vector; ^##^*P* < 0.01 vs. mimic NC + CASC7 or Vector + miR-34a-5p mimic. Data are expressed as the mean ± standard deviation (*n* = 3)

Finally, we determined whether CASC7 regulates PTC cell behavior through sponging miR-34a-5p. In contrast to CASC7 overexpression, miR-34a-5p mimic transfection significantly promoted cell proliferation (Fig. 5a) and migration (Fig. 5c), whereas inhibited cell apoptosis (Fig. 5b) in both K1 and TPC-1 cells. More importantly, the CASC7 overexpression-mediated inhibition of cell proliferation and migration and promotion of cell apoptosis could be abated following introduction of miR-34a-5p mimic (Fig. 5a–c).

## Discussion

In the present study, we investigated the expression of CASC7, miR-34a-5p and TP73 in PTC tissues. CASC7 and TP73 expressions were found to be significantly downregulated, whereas miR-34a-5p was upregulated in PTC tissues. Furthermore, we explored their interaction and role in regulating PTC cell behavior. Our results showed that CASC7 acted as a sponge of miR-34a-5p to upregulate the expression of tumor-suppressive TP73, and thereby inhibited PTC cell proliferation and migration and induced PTC cell apoptosis.

CASC7 has been shown to be involved in several diseases, such as asthma and myocardial ischemia–reperfusion injury [17, 18]. With regard to cancer, CASC7 has been reported in glioma and colorectal cancer where CASC7 exerted tumor-suppressive roles [15, 16]. However, the expression and function of CASC7 in other cancers remains far from being elaborated. To our knowledge, this study is the first to show the downregulated expression and tumor-suppressive role of CASC7 in PTC. Our results also revelaed that CASC7 expression showed a downward trend with PTC stage progression. However, further investigation was required to elucidate the association of CASC7 expression with clinicopathological features of PTC.

MiRNAs are small noncoding RNA molecules (21 ∼ 23 nucleotides) that play an important role in regulating various cellular processes such as cell proliferation, differentiation, and apoptosis [19]. A study by Wang et al*.* revealed that miR-34a-5p expression is significantly higher in PTC plasma samples and tissues relative to healthy controls [20], indicating that miR-34a-5p might play a crucil role in PTC progression. MiR-34a-5p exerts tumor-suppressive or oncogenic role in cancer [21]. For example, miR-34a-5p overexpression has been demonstrated to inhibit ovarian cancer cell proliferation and trigger apoptosis [22]. In contrast, Jing et al. reported that miR-34a-5p plays a tumor-promoting role in clear cell renal cell carcinoma [23]. Consistent with the oncogenic role, our results showed that miR-34a-5p mimic promoted cell proliferation and migration, and inhibited cell apoptosis in both K1 and TPC-1 cells. To our knowledge, this study is the first to demonstrate the oncogenic role of miR-34a-5p in PTC cells.

It has been well accepted that lncRNAs can function as competing endogenous RNAs (ceRNAs) or as molecular sponges in modulating the expression and biological functions of miRNAs in various cellular context [24, 25]. Increasing lncRNAs have been reported to participate in the progression of PTC through the ceRNA mechanism [14, 26]. For instance, lncRNA TUG1 (taurine upregulated gene 1) contributed to the progression of PTC cells through regulating miR-145/zinc finger E-box binding homeobox 1 (ZEB1) pathway [27]. Similarly, CASC7 can also regulate cancer development through acting as a ceRNA [15]. The TP73 gene, located on chromosome 1p36.3, encodes a product that shares significant structural homology with the tumor suppressor TP53 [28]. Abundant studies have characterized TP73 as a tumor-suppressive gene [28–30]. Using bioinformatics analysis and luciferase reporter assay, we confirmed TP73 as a direct target of miR-34a-5p. Furthermore, CASC7 can bind to miR-34a-5p and inhibit miR-34a-5p expression in PTC cells. Importantly, the miR-34a-5p-mediated targeted suppression of TP73 and promotion of PTC cell growth were abrogated when CASC7 was overexpressed in PTC cells. Thus, we conclude that CASC7 acts as a ceRNA of miR-34a-5p, leading to the derepression of the miR-34a-5p target TP73, and eventually regulated PTC cell behavior.

## Conclusion

In summary, the findings in the present study demonstrate that CASC7 functions as a ceRNA of miR-34a-5p by sponging miR-34a-5p to upregulate its target TP73, thereby inhibiting PTC cell proliferation and migration and inducing PTC cell apoptosis. Our study extends the knowledge on the mechanism underlying PTC progression and provides a new perspective for CASC7-directed drug targets for PTC.

### Supplementary Information

**Additional file 1: Figure S1.** qRT-PCR analysis of CASC7 expression in PTC tissues (PTC-I n=14; PTC-II n=4; PTC-III n=9; PTC-IV n=3) and adjacent normal tissues (n=30). ^**^P<0.01 vs. Tumor-adjacent tissues.

**Additional file 2: Figure S2.** **(A, C)** TP73 mRNA expression determined by qRT-PCR analysis, and **(B, D)** TP73 protein level determined by western blot in human PTC cell lines (K1 and TPC-1) transfected with the sh-CASC7 or sh-NC. ^**^P<0.01 vs. sh-NC. Data are expressed as the mean ± standard deviation (n=3).

## Data Availability

The datasets used in the current study are available from the corresponding author on reasonable request.

## Abbreviations

TC: Thyroid cancer
PTC: Papillary thyroid cancer
lncRNA: Long noncoding RNA
CASC7: Cancer susceptibility candidate 7
miR: MicroRNA
DMEM: Dulbecco’s modified Eagle’s medium
FBS: Fetal bovine serum
NC: Negative control
shRNA: Short hairpin RNA
qRT-PCR: Quantitative real-time PCR
TP73: Tumor protein P73
WT: Wild type
Mut: Mutant
ceRNA: Competing endogenous RNA
TUG1: Taurine upregulated gene 1
