# Supplementary figures and images for: Long noncoding RNA CASC7 inhibits the proliferation and migration of papillary thyroid cancer cells by inhibiting miR-34a-5p
Source: J Physiol Sci. 2021 Mar 11;71:9. doi: 10.1186/s12576-021-00793-2 (PMC10718045; doi:10.1186/s12576-021-00793-2)

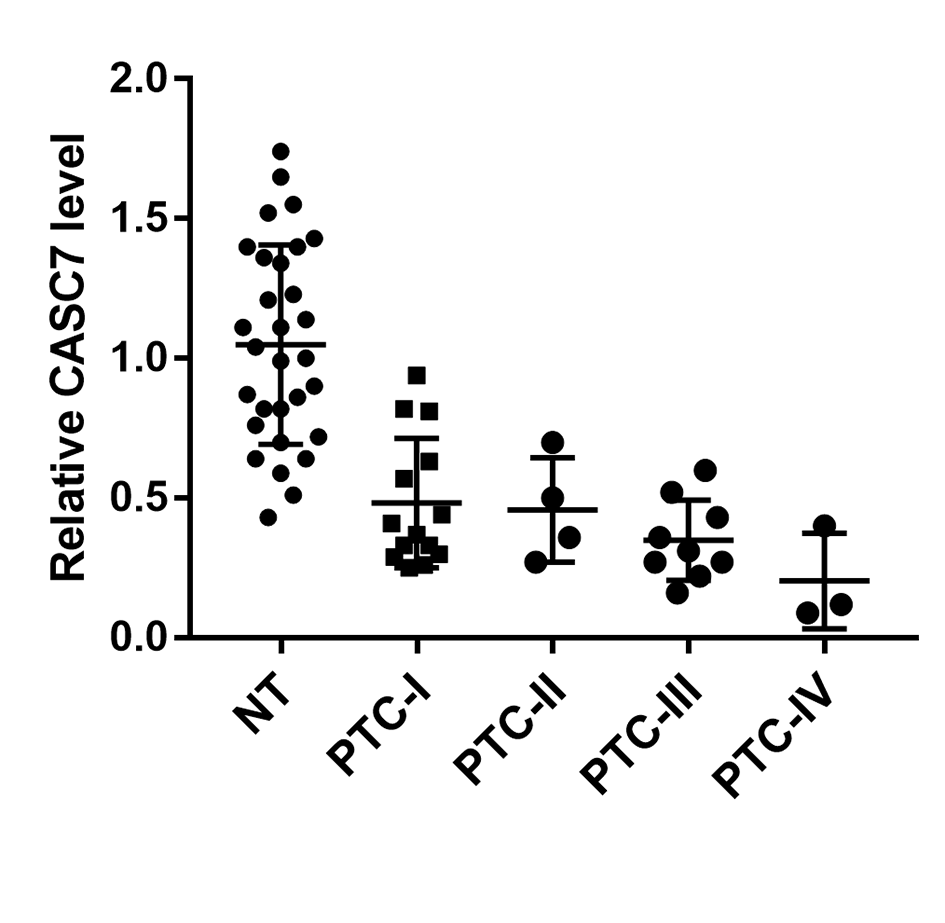

Supplement: Supplementary file 1 — Additional file 1: Figure S1. qRT-PCR analysis of CASC7 expression in PTC tissues (PTC-I n=14; PTC-II n=4; PTC-III n=9; PTC-IV n=3) and adjacent normal tissues (n=30). **P<0.01 vs. Tumor-adjacent tissues. [file 12576_2021_793_MOESM1_ESM.tif]

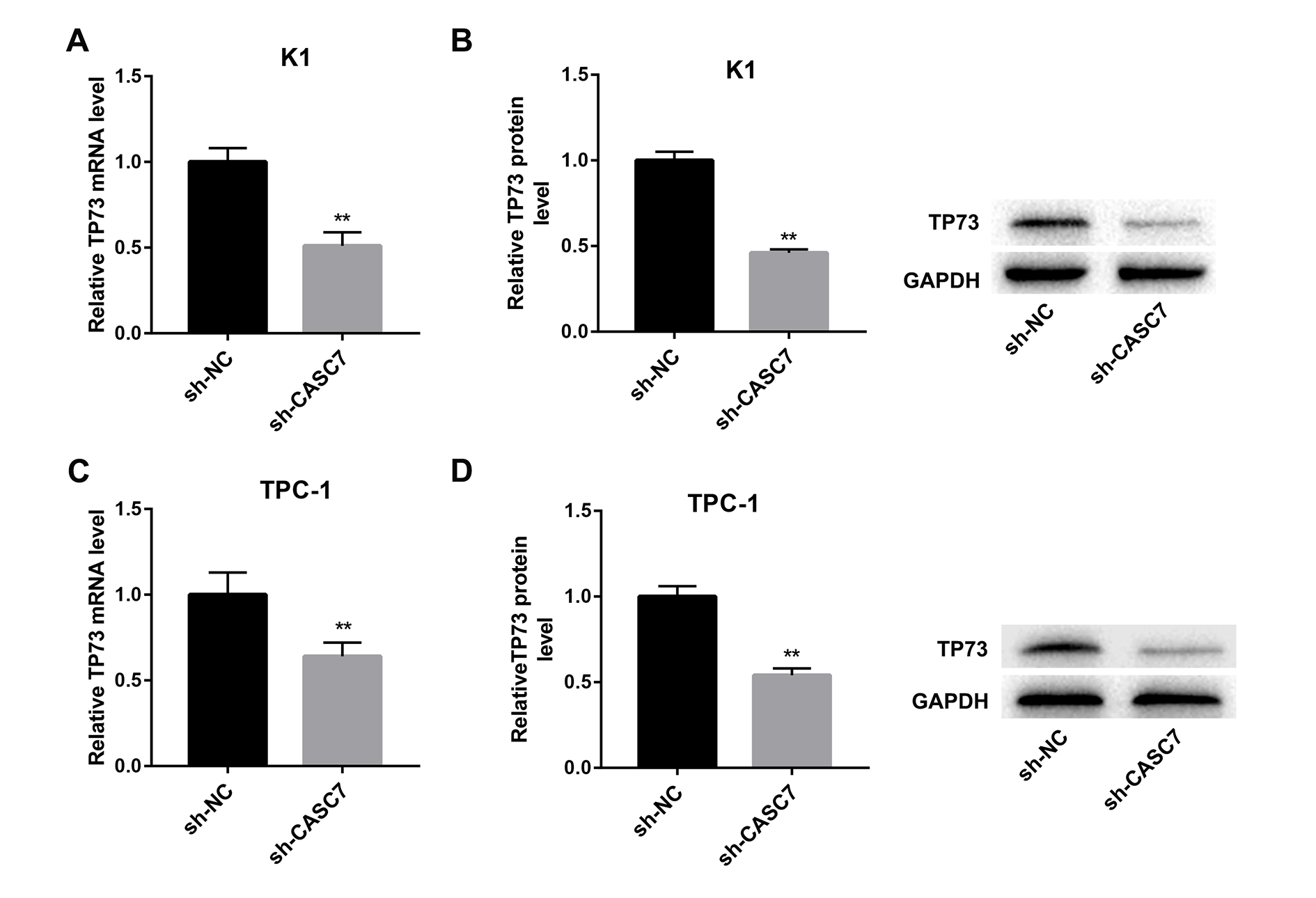

Supplement: Supplementary file 2 — Additional file 2: Figure S2. (A, C) TP73 mRNA expression determined by qRT-PCR analysis, and (B, D) TP73 protein level determined by western blot in human PTC cell lines (K1 and TPC-1) transfected with the sh-CASC7 or sh-NC. **P<0.01 vs. sh-NC. Data are expressed as the mean ± standard deviation (n=3). [file 12576_2021_793_MOESM2_ESM.tif]
